# Urinary TNF-α as a potential biomarker for chronic primary low back pain

**DOI:** 10.3389/fnint.2023.1207666

**Published:** 2023-06-28

**Authors:** Carlos Gevers-Montoro, Mariana Puente-Tobares, Aléxiane Monréal, Francisco Miguel Conesa-Buendía, Mathieu Piché, Arantxa Ortega-De Mues

**Affiliations:** ^1^Madrid College of Chiropractic – RCU María Cristina, Madrid, Spain; ^2^Department of Anatomy, Université du Québec à Trois-Rivières, Trois-Rivières, QC, Canada; ^3^CogNAC Research Group, Université du Québec à Trois-Rivières, Trois-Rivières, QC, Canada; ^4^Instituto de Investigación Sanitaria, Fundación Jiménez Díaz, Madrid, Spain; ^5^Fujitega Research Foundation, Madrid, Spain

**Keywords:** TNF-alpha, urine biomarkers, chronic pain, instrument-assisted spinal manipulation, pain trajectories, back pain

## Abstract

**Introduction:**

Over two thirds of individuals with low back pain (LBP) may experience recurrent or persistent symptoms in the long term. Yet, current data do not allow to predict who will develop chronic low back pain and who will recover from an acute episode. Elevated serum levels of the proinflammatory cytokine tumor necrosis factor-α (TNF-α) have been associated with poor recovery and persistent pain following an acute episode of LBP. Inflammatory cytokines may also mediate mechanisms involved in nociplastic pain, and thus, have significant implications in chronic primary low back pain (CPLBP).

**Methods:**

This study aimed to investigate the potential of urinary TNF-α levels for predicting outcomes and characterizing clinical features of CPLBP patients. Twenty-four patients with CPLBP and 24 sex- and age-matched asymptomatic controls were recruited. Urinary TNF-α concentrations were measured at baseline and after 4 weeks, during which CPLBP patients underwent spinal manipulative therapy (SMT).

**Results:**

Concentrations of TNF-α were found to be elevated in baseline urine samples of CPLBP patients compared to asymptomatic controls. Moreover, these values differed among patients depending on their pain trajectory. Patients with persistent pain showed higher levels of TNF-α, when compared to those with episodic CPLBP. Furthermore, baseline TNF-α concentrations and their changes after 4 weeks predicted alterations in pain intensity and disability following SMT in patients with CPLBP.

**Discussion:**

These findings warrant further research on the potential use of urinary TNF-α concentrations as a prognostic biomarker for CPLBP.

## Introduction

A large proportion of the general population will be afflicted with low back pain (LBP) at some point in their lifetime (Hoy et al., 2012; Vlaeyen et al., 2018), particularly in working age groups (Hartvigsen et al., 2018). It is likely that more than half a billion individuals suffer from LBP at any time point (Wu et al., 2020), some on an ongoing basis (Hoy et al., 2012). The exact proportion of patients who develop chronic LBP is currently unknown, but recent estimates suggest that one to two thirds of people seeking care for acute LBP may eventually experience recurrence or persistence of symptoms (Itz et al., 2013; da Silva et al., 2017).

Aiming to identify those who recover from an acute episode of LBP and those who do not, efforts have been directed toward investigating the patients’ trajectories (Axen and Leboeuf-Yde, 2013; Kongsted et al., 2016). Most patients exhibit symptom trajectories characterized by either fluctuating or episodic LBP (Kongsted et al., 2017). Identifying the factors that influence distinct trajectories can enhance our ability to predict and categorize the course of LBP in individual patients. The severity of pain trajectories generally shows positive associations with female gender, history of LBP, the presence of leg pain, and comorbidities such as depression (Kongsted et al., 2015, 2016). In addition, LBP episodes and trajectories are strongly influenced by inflammation (Klyne et al., 2017).

Several potential inflammatory biomarkers have been identified in the context of LBP (Khan et al., 2017; Morris et al., 2020). Of these, the proinflammatory cytokine Tumor Necrosis Factor-alpha (TNF-α) has been associated with poor long-term recovery from acute episodes of LBP and symptom persistence (Klyne et al., 2017, 2022; Queiroz et al., 2017; Klyne and Hodges, 2020; Morris et al., 2020). Moreover, TNF-α plays a significant role in the development and maintenance of central sensitization (Andrade et al., 2011; Ji et al., 2018; Vergne-Salle and Bertin, 2021), one of the main neurophysiological mechanisms underpinning nociplastic pain conditions (Nijs et al., 2021; Treede et al., 2022). The presence of nociplastic mechanisms in LBP is highly suggestive of chronic primary low back pain (CPLBP), previously classified as non-specific (Kosek et al., 2021; Treede et al., 2022). CPLBP is chronic LBP of an unexplained etiology that is not fully attributable to either nociceptive or neuropathic mechanisms. Identifying biomarkers for CPLBP remains an unresolved challenge, which could prove extremely useful to understand the pathogenesis, prognosis and treatment response of individual patients or patient subgroups (Davis et al., 2020).

It has been proposed that non-pharmacological approaches, such as manual therapy, may modulate inflammatory responses and nociplastic pain mechanisms in patients with CPLBP, however, this remains unclear (Licciardone et al., 2012; Lima et al., 2020; Gevers-Montoro et al., 2021). Elevated *in vitro* production of TNF-α in whole blood cultures of patients with CPLBP was significantly reduced after a period of spinal manipulative therapy (SMT) (Teodorczyk-Injeyan et al., 2021). These findings were recently replicated in urine samples of individuals with CPLBP (Gevers-Montoro et al., 2022), suggesting that TNF-α levels may reflect clinical outcomes or mechanisms relevant to their prognosis. A better understanding of the role TNF-α plays in persons with CPLBP could have the potential to inform mechanisms involved in the course and recovery from CPLBP, in particular, for patients undergoing SMT.

Therefore, the aim of this study was to assess the predictive value of urinary concentrations of TNF-α for outcomes and clinical characteristics in patients with CPLBP. First, we aimed to confirm that baseline urinary concentrations of TNF-α were elevated in patients with CPLBP compared with age-sex matched pain-free controls. Secondly, we compared changes in urinary concentrations of TNF-α over 4 weeks, during which patients received standardized SMT and controls received no intervention. We hypothesized that TNF-α concentrations would decrease in patients with CPLBP, approaching values observed in controls. Thirdly, we examined the predictive value of urinary TNF-α concentrations for clinical characteristics and outcomes in patents with CPLBP that received SMT. We hypothesized that urinary TNF-α concentrations may be used as a biomarker to discriminate patients with CPLBP according to their pain trajectory and to predict clinical recovery.

## Materials and methods

### Study design and ethical approval

This was a prospective case-control study with longitudinal follow-up, assessing the predictive value of urinary TNF-α concentrations for baseline characteristics and clinical evolution of CPLBP patients undergoing chiropractic instrument-assisted SMT. The study protocol was approved by the Madrid College of Chiropractic Research subcommittee (San Lorenzo de El Escorial, Madrid, Spain) and the Fundación Jiménez Díaz Hospital Clinical Research Ethics Committee (Madrid, Spain). The study was conducted between January 2018 and December 2022 at the Madrid College of Chiropractic Outpatient Clinic. All experimental procedures conformed to the standards set by the latest revision of the Declaration of Helsinki.

### Participant recruitment

Patients were recruited from the population visiting the outpatient clinic for an initial consultation with a chief complaint of CPLBP. Patients seeking care for symptoms of LBP were screened for inclusion and exclusion criteria by performing a complete case history and physical examination, following routine protocols from the outpatient clinic. The inclusion criteria were: being between 18 and 80 years of age and presenting a chief complaint of persistent or recurrent pain ≥3 months, in any anatomical location included between the lower margin of the 12th rib to the lower gluteal folds, with or without referring to the lower limbs (Vlaeyen et al., 2018). The exclusion criteria were the following: detection of a specific pathology as the cause for the LBP, including evidence for pain of neuropathic origin, such as radicular symptoms, as this is considered chronic secondary LBP (Nicholas et al., 2019; Kosek et al., 2021); presence of chronic pain of higher perceived severity than LBP in any other body region; previous diagnosis of an inflammatory or rheumatic condition (e.g., inflammatory spondyloarthropathies); any contraindication to SMT (vertebral instability, history of any spine or pelvis fracture or surgery, namely spinal fusion or discectomy); having received any form of manual therapy to the spine in the previous 2 years; current use of prescribed pain medication, with the exception of non-steroidal anti-inflammatory drugs and over-the-counter analgesics; and pregnancy. Exclusion criteria allowed to identify a population with a diagnosis of chronic primary LBP (Nicholas et al., 2019). Once the diagnosis was confirmed, patients deemed eligible were informed about the study and were offered to participate. Patients accepting to participate read and signed an informed consent form before initiating treatment and collecting samples. Patients declining participation continued their regular course of care at the clinic without prejudice.

A cohort of pain-free controls matched by sex and age to the patient cohort was enrolled to serve as a reference for the levels in inflammatory cytokines that were collected and assessed from the patient cohort. Individuals eligible for the pain-free cohort were to meet the following criteria: aged between 18 and 80 years old, without acute or chronic pain symptoms or diagnoses, and without a current or prior diagnosis of any systemic, inflammatory, neurological, or psychiatric conditions. Pain-free individuals accepting to participate read and signed an informed consent form before urine sample collection. Informed consent was also obtained from all subjects for publication of identifying information/images in an online open-access publication.

As the first aim of the study was to assess urinary levels of TNF-α in patients with CPLBP before and after receiving SMT, the targeted sample size was based on a previous observational study reporting elevated levels of urinary TNF-α that decreased after exposure to chiropractic care mainly based on SMT (Gevers-Montoro et al., 2022). Considering a more homogenous CPLBP population and more standardized care for the current study, similar or larger effect sizes were expected. Thus, power calculations were based on an effect size of Cohen’s *d* = 0.6, an alpha of 0.05 and a statistical power of 0.8 for a mixed model assessing both within- and between-subject interactions. The required sample size was of 24 participants per group [G*Power version 3.1.9.6 (Faul et al., 2007)], 24 patients with CPLBP and an identical number of pain-free controls matched for sex and age.

### Treatment procedures

**FIGURE 1:**
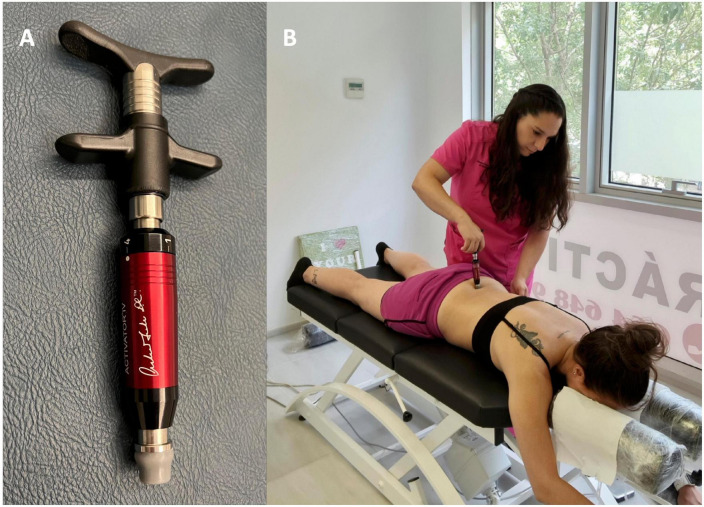
An activator IV^®^ instrument **(A)**. A chiropractor applying a spinal manipulation to the lumbar spine using the Activator IV^®^ instrument **(B)**.

Patients recruited for the study were scheduled for the first treatment session 24−48 h following the initial examination. They underwent a standardized unimodal care plan, based exclusively on the delivery of instrument-assisted SMT by a chiropractor, twice a week for a total duration of 4 weeks. Frequency of care was standardized in order to reflect clinical practice (Schneider et al., 2015) and comply with clinical practice guidelines (Globe et al., 2016). Re-assessment took place within 24 h of the eighth and last session. Treatment consisted in the delivery of high-velocity low amplitude manipulations with the assistance of the Activator IV^®^ mechanical device (FDA approval # K003185, Manufacturer: Activator Methods International Ltd., Phoenix, AZ, USA). This instrument is a hand-held device (Figure 1A) containing a spring-loaded mechanism that delivers a mechanical impulse with four different settings. The use of an instrument-assisted protocol of SMT was preferred in order to standardize treatment protocols and reduce variability in force application (Kawchuk et al., 2006; Descarreaux et al., 2013). This would allow to determine whether the site, number and magnitude of force applications had any impact on the primary outcome. To date, it remains unclear whether the dosage or the site of force application have an impact on clinical or neurophysiological outcomes (Pasquier et al., 2019; Nim et al., 2021). Settings 1−3 were used in the cervical and thoracic spines with peak forces ranging from 115 to 123 N, while setting 4 was used in the lumbopelvic spine (including T12) and delivers forces around 211 N, all force applications with a duration of ∼ 5 ms (Colloca et al., 2005). Manipulations were applied in the prone position (Figure 1B) to segmental levels determined by the Activator Methods protocol and manual palpation (Fuhr et al., 1996; Schneider et al., 2015). Upon completing the last treatment session, a physical re-evaluation of the patient was performed, including evaluation of the outcome measures, described below.

### Primary outcome: urinary levels of TNF-α

Patients and controls provided a baseline urine sample of the first morning micturition on the day they received their first SMT session (patients) or on the day after being recruited (controls). All participants were instructed to store their urine samples in the refrigerator (∼ 4°C) immediately after collection and until visiting the clinic. Once urine samples were collected, they were immediately aliquoted and stored in a container at −20°C. The procedure was identical for the follow-up sample, which was collected 4 weeks after the baseline sample collection. For patients, this corresponded to the day after the eighth and final SMT session. All participants were requested to refrain from taking any anti-inflammatory medication within 24 h of the dates when both samples were collected.

Urinary TNF-α concentrations were measured in duplicate by using specific commercial sandwich enzyme-linked immunosorbent assay (ELISA) following manufacturer’s recommendations (Cloud-Clone Corp., Houston, TX, USA) (Sirera et al., 2003). Urinary concentrations of TNF-α (pg/ml) and creatinine (mg/dl) were assessed for each sample, following the same method that was reported previously (Gevers-Montoro et al., 2022). The ratio of urinary TNF-α to urinary creatinine in pg/mg was calculated to correct for changes in urine volume (Ortega et al., 2019). All statistical analyses and figures used and display the corrected values in pg/mg.

### Secondary outcomes: clinical outcome measures

Clinical variables describing comorbidities, CPLBP duration and trajectories were collected in the initial clinical interview. The presence of comorbidities included chronic non-painful conditions and pain affecting other body sites. Duration since the onset of the first episode was recorded in years. CPLBP trajectories were classified as either “ongoing,” “fluctuating,” or “episodic” (independent of severity), according to suggested criteria (Kongsted et al., 2017). Episodic CPLBP was defined as pain occurring with pain-free periods of at least 4 weeks. The trajectory was classified as fluctuating when patients recalled variations of 2 or more points in an 11-point numerical rating scale (NRS), without pain-free periods of 4 weeks or longer. Finally, ongoing pain implied a relatively stable pain intensity ( ± 1 point in the NRS) present at least 4 days a week (Kongsted et al., 2017). Available data suggest that patients may recall their recent LBP trajectory (for up to 6 months) with an acceptable degree of precision (Hestbaek et al., 2019). These variables were used to identify potential patient subgroups with different levels in urinary TNF-α.

**FIGURE 2:**
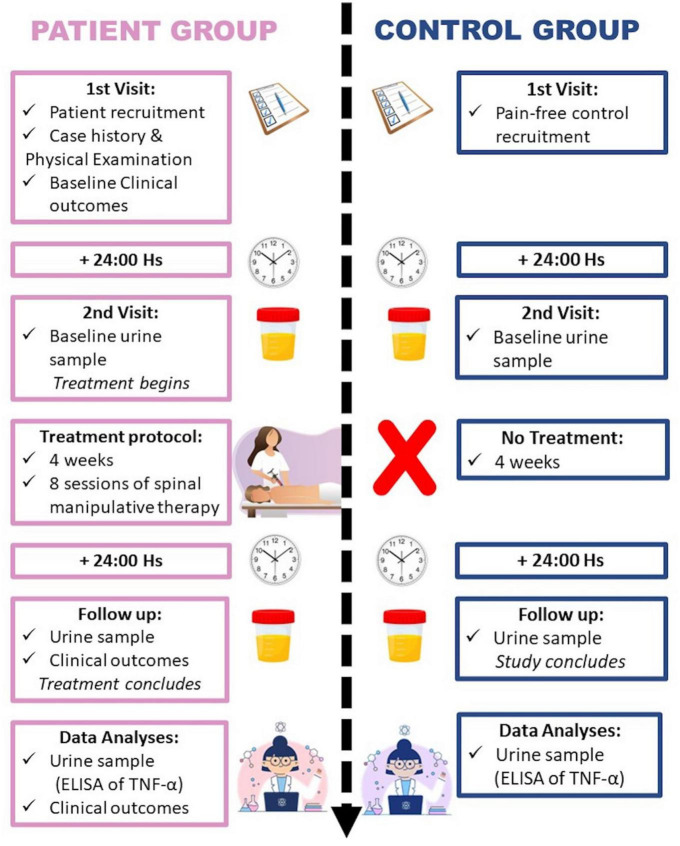
Flowchart representation of the study protocol.

To examine changes in pain intensity, patients reported their current pain intensity in a NRS ranging from 0 to 10, anchored by two verbal descriptors. The anchor 0 indicated “no pain,” while 10 indicated “worst pain imaginable.” Functional impairment caused by CPLBP was measured by means of the Oswestry LBP Disability Index (ODI) questionnaire, a scale that is widely used in LBP research (Fairbank and Pynsent, 2000). Its validated version in Spanish has good to excellent reliability (Alcántara-Bumbiedro et al., 2006). The ODI score ranges from 0 to 50, with higher numbers representing higher levels of self-reported disability. It consists of ten questions with six possible answers that are graded from 0 to 5 points, based upon the severity of self-perceived disability for each of the activities of daily living. Both pain intensity and disability were measured at the baseline session (before initiating care) and within 24 h of the last treatment session (Figure 2).

### Statistical analysis

All statistical analyses were conducted using JASP v0.16.4 (JASP Team, 2022) and Jamovi v2.3.21 (The Jamovi Project, 2022). Normality distribution was assessed for baseline quantitative data by means of Shapiro-Wilk tests and homoscedasticity with Levene’s tests. A *p*-value threshold of 0.05 was considered statistically significant for all analyses. Values presented in the results section represent mean ± standard deviation. TNF-α data were not normally distributed, therefore, to test *a priori* hypotheses, baseline urinary TNF-α concentrations were compared between both groups by means of a Welch’s *t*-test due to heteroscedasticity and non-normal distribution. Further, changes in TNF-α before and after the 4 weeks were analyzed using a linear mixed model (Schielzeth et al., 2020), with time (repeated measures; follow-up–baseline), group (patients vs. controls), and the time × group interaction as fixed effects, and participants as random effects (intercept modeled). Pain intensity ratings and disability scores at baseline and after eight sessions of SMT were compared using paired *t*-tests for exploratory purposes.

To identify potential differences in urinary concentrations of TNF-α at baseline, according to sex, pain trajectories and the presence of comorbidities, Mann–Whitney tests or Kruskal–Wallis analyses of variance (ANOVA) were conducted using these categorical variables as grouping variables. Significant ANOVA effects were decomposed using Dwass-Steel-Critchlow-Fligner pairwise comparisons. In addition, Spearman rank correlation coefficients were calculated to examine the associations between baseline values of TNF-α, the number of years with CPLBP, pain intensity and disability. To explore the predictive value of urinary TNF-α, baseline, follow-up, and percent-changes in TNF-α values were assessed as predictors in simple regression models with follow-up and percent change values in pain intensity and disability as dependent outcomes, for which estimates were obtained using 5000 bootstrap replications.

A supplementary exploratory analysis was conducted to identify associations with SM dosage and target site. Spearman correlations were assessed between changes and follow-up values of TNF-α, and the total number of SM applied to low back segments (sacroiliac joints, L5, L4, L2, and T12), to the lumbopelvic area and to the whole body.

## Results

For the thirty-nine patients that were screened for eligibility, twenty-four met the selection criteria and were included in the study. Eighteen patients were women and six were men, with a mean age of 53.9 ± 10 years, and a mean of 11.5 ± 8.3 years with CPLBP (Table 1). The fifteen patients that were excluded from the study presented pain of neuropathic origin, were taking opioid medication, presented complaint of neck pain of similar severity, received chiropractic care or manipulation recently, or presented with a diagnosis of spondyloarthropathy. Twenty-four pain-free controls were recruited to match the CPLBP patients, with the same proportion of women and men as the patient group, and a mean age of 53.6 ± 9 years (Table 1).

**TABLE 1:** Baseline demographic and clinical data of participants in the study.

Baseline characteristic	Patient group	Control group
Participants, *n*	24	24
Sex (women), *n* (%)	18 (75)	18 (75)
Mean age, years (SD)	53.9 (10.0)	53.9 (8.8)
Smokes (Yes), *n* (%)	0 (0)	4 (17)
Mean TNF-α values (pg/mg), (SD)	3.7 (4.6)	0.3 (1.4)
**Chronic low back pain characteristics**		
Mean pain intensity (0−10), (SD)	5.8 (1.7)	−
Mean disability score (0−50), (SD)	14.7 (7.0)	−
Mean years with pain, (SD)	11.5 (8.3)	−
**Pain trajectory, *n* (%)**		
Ongoing	9 (37)	−
Fluctuating	11 (46)	−
Episodic	4 (17)	−
Comorbidities (Yes), *n* (%)	14 (58)	−
Taking NSAIDs (Yes), *n* (%)	8 (33)	−

SD, standard deviation; NSAIDSs, non-steroidal anti-inflammatory drugs.

### Urinary levels of TNF-α in patients and pain-free controls

**FIGURE 3:**
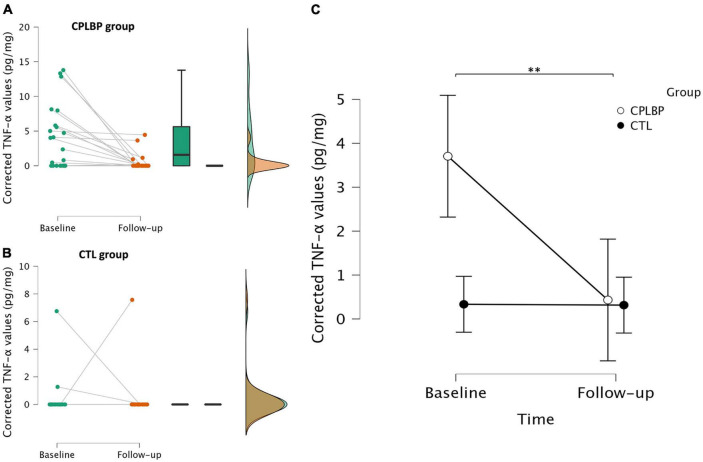
Raincloud plots (Allen et al., 2019) combining a cloud of points with a box plot and a one-sided violin plot of the distribution of urinary concentrations of TNF-α corrected for volume (using urine creatinine) at baseline and follow-up for the control **(A)** and patient **(B)** groups. Individual dots represent individual participant values and the lines within the box plot represent the median. Descriptive plot of the mean urinary concentrations of TNF-α corrected for volume at baseline and follow-up for the control and patient groups. Bars represent 95% confidence intervals **(C)**. **p < 0.01 (significance level for the time × group interaction). CTL, control group; CPLBP, chronic low back pain group.

The mean baseline urinary concentration of TNF-α corrected for urine volume was 3.7 ± 4.6 pg/mg in the patient group and 0.3 ± 1.4 pg/mg in the control group (see Figure 3 and Table 1). The mean difference of 3.37 pg/mg [1.37−5.38 pg/mg, 95% confidence interval (CI)] was statistically significant (*p* = 0.002, *d* = 0.99). Follow-up values were 0.4 ± 1.2 pg/mg for the CPLBP group and 0.3 ± 1.6 pg/mg for the control group. The estimated difference between group means over time was of −3.25 pg/mg (−5.35 to −1.16, 95% CI), which was statistically significant (interaction: *F*_1,92_ = 9.5, *p* = 0.003, *η^2^p* = 0.11, Figure 3C). As some patients (*n* = 14) were taking non-steroidal anti-inflammatory drugs, this variable was introduced as a categorical covariate in the mixed model to examine the potential confound. The results remained unchanged (interaction: *p* = 0.003).

### Clinical outcomes in patients with CPLBP

**FIGURE 4:**
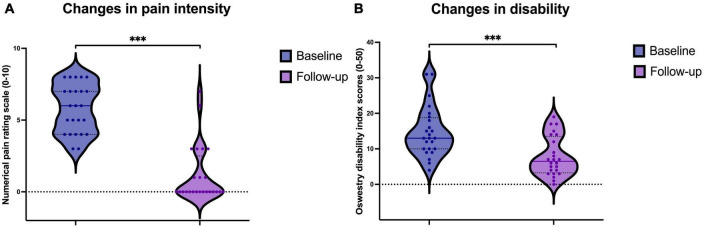
Violin plot of the distribution of **(A)** Pain intensity ratings in the numerical rating scale (NRS) from 0 to 10 and **(B)** disability scores measured with the Oswestry Disability Index, both at baseline and follow-up. Individual dots represent individual patient values. The continuous line represents the median and dotted lines represent 25th and 75th quartiles. ***p < 0.001.

Significant reductions were observed in clinical outcomes following the eight sessions of SMT in the patient group. Pain intensity was reduced in 4.6 ± 2.1 points in the 0−10 NRS scale, *p* < 0.001, *d* = 2.2 (Figure 4A; Table 1). Furthermore, the degree of disability caused by CPLBP was also reduced by 6.9 ± 5.5 points in the ODI 0−50 scale, *p* < 0.001, *d* = 1.24 (Figure 4B; Table 1).

### Differences in TNF-α values at baseline by grouping variables in patients with CPLBP

Analyses were conducted to examine differences in TNF-α values at baseline according to sex, pain trajectory, and the presence of comorbidities in the patients with CPLBP. Baseline concentrations of TNF-α were significantly different between subgroups of patients with different pain trajectories (χ^2^ = 9.28, *p* = 0.01, df = 2, ε^2^ = 0.4). Baseline values were then calculated separately for patients with ongoing (6.6 ± 4.6 pg/mg, *n* = 9), fluctuating (2.7 ± 4.2 pg/mg, *n* = 11) and episodic (0 pg/mg, *n* = 4) CPLBP. Pairwise comparisons revealed that ongoing pain trajectory levels were significantly different from episodic (*p* = 0.03), but not fluctuating (*p* = 0.1). TNF-α levels did not significantly differ between fluctuating and episodic CPLBP (*p* = 0.12). Moreover, baseline TNF-α did not differ by sex (Mann-Whitney *U* = 32.0, *p* = 0.1). Fourteen patients presented comorbid conditions with CPLBP (see Table 1). Comorbidities were cardiovascular disease (*n* = 3), neck pain (*n* = 3), depression (*n* = 2), full spine pain (*n* = 2), headaches (*n* = 2), type II diabetes (*n* = 1), anxiety (*n* = 1), carpal tunnel syndrome (*n* = 1) and plantar fasciitis (*n* = 1). There were no differences in TNF-α levels based on the presence of comorbidities (Mann–Whitney *U* = 69.0, *p* = 1.0).

### Associations with TNF-α values at baseline in patients with CPLBP

**FIGURE 5:**
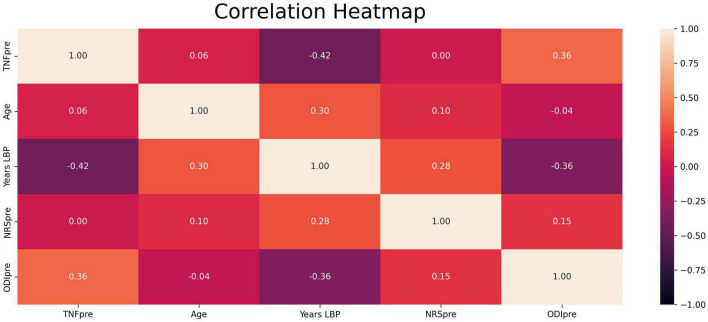
Heatmap of Spearman rank correlations between all variables of interest. Values in the boxes represent Spearman coefficient ρ. “TNFpre”: Baseline levels of TNF-α; “Age”: Age in years; “Years LBP”: years since onset of low back pain; “NRSpre”: Baseline pain intensity ratings; “ODIpre”: Baseline disability scores; “ODIpost”: Follow-up disability scores; “%ODI”: Percent changes disability scores.

Spearman rank correlation coefficients revealed only one significant (negative) association between the number of years with CPLBP and baseline TNF-α (ρ = −0.42, *p* = 0.04, Figure 5). This association, however, was not significant when correcting for the number of comparisons.

### Urinary TNF-α as a predictor of clinical outcomes in patients with CPLBP

Simple regression analyses revealed that baseline TNF-α values explained 20.7% of the variance in changes in pain intensity (*F* = 5.8, *p* = 0.03), however, baseline TNF-α only marginally predicted percent changes in pain intensity (β = −0.45; *p* = 0.05). Follow-up pain intensity ratings were not predicted by baseline urinary TNF-α (β = 0.24; *p* = 0.4). Percent changes in disability could not be predicted by baseline TNF-α (β = −0.25; *p* = 0.1), but follow-up values in disability could (β = 0.64; *p* = 0.002). The latter model was significant as well (*F* = 15.2, *p* < 0.001), 38.1% of the variance in follow-up ODI scores were explained by baseline TNF-α.

Regression analyses with the percent change in TNF-α as a predictor showed that 65% and 33% of the variance in pain intensity and disability percent changes, respectively, could be explained by fluctuations in TNF-α (β = 0.81; *p* < 0.001 and β = 0.58; *p* = 0.003, respectively). Both models were also significant: *F* = 41.1, *p* < 0.001 for pain intensity and *F* = 11.0, *p* = 0.003 for disability. However, changes in TNF-α did not predict follow-up values in pain intensity (β = −0.02; *p* = 0.8) nor disability (β = −0.27; *p* = 0.1).

### Associations between the segments targeted by SM and TNF-α in patients with CPLBP

Associations between follow-up values and percent changes in TNF-α with the number of SM received were examined with exploratory purposes. However, no strong or significant associations were detected, with the exception of a marginal association between the total number of lumbopelvic manipulations and changes in TNF-α (ρ = −0.40, *p* = 0.049). See Supplementary Figure 1 for the correlation heatmap.

## Discussion

The present study corroborates previous reports of elevated levels of TNF-α in both serum and urine samples of patients with CPLBP (Teodorczyk-Injeyan et al., 2019; Morris et al., 2020; Gevers-Montoro et al., 2022). Furthermore, in this cohort of patients, urinary concentrations of this pro-inflammatory cytokine were reduced after SMT, compared to values in matched pain-free controls. Baseline levels in urinary TNF-α discriminated patients according to their CPLBP trajectory, the highest levels being measured in patients with unremitting pain. In turn, baseline TNF-α concentrations and their fluctuations predicted changes in both pain intensity and disability scores.

The present findings are consistent with prior research suggesting that patients with CPLBP have elevated concentrations of TNF-α in urine (Gevers-Montoro et al., 2022). Moreover, this study shows that urinary TNF-α may accurately discriminate patients with CPLBP from age- and sex-matched asymptomatic individuals. In the absence of inflammation, both serum and urinary levels of TNF-α are presumed to approach zero, with minimal fluctuations (McLaughlin et al., 1991; Feghali and Wright, 1997; Biancotto et al., 2013; Wang et al., 2016; Moledina et al., 2019). Levels detected in an asymptomatic population in this study are consistent with suggested reference values of 0.4 ± 0.8 pg/mg (Gevers-Montoro et al., 2022). Moreover, the absence of significant fluctuations over a 4-week period in pain-free individuals was confirmed. Notably, baseline values differed significantly among patients with distinct pain trajectories, specifically between those with “ongoing” compared to “episodic” pain. Patients categorized as “ongoing” generally exhibited higher urinary levels of TNF-α (6.6 ± 4.6 pg/mg), followed by patients classified as “fluctuating” (2.7 ± 4.2 pg/mg). In contrast, patients with “episodic” CPLBP had undetectable levels of this cytokine, rendering them biochemically indistinguishable from healthy individuals in this regard. This suggests that different mechanisms may underlie different pain trajectories. A previous assessment of urinary TNF-α values in CPLBP patients showed mean values of 6.0 ± 7.0 pg/mg, in a cohort where 75% of patients were classified as “ongoing” (Gevers-Montoro et al., 2022), which is consistent with data from the current study.

The results presented in this study indicate that TNF-α may emerge as a potential patient stratification biomarker, which is crucial in health conditions with heterogeneous pathophysiology, such as CPLBP (Davis et al., 2020). Urinary TNF-α could help discriminate patients with CPLBP according to their pain trajectory. Specifically, patients experiencing persistent pain (whether ongoing or fluctuating, but not remitting), may be better identified by this biomarker. Evidence from systematic reviews highlights an association between TNF-α and the presence of CPLBP (Khan et al., 2017; van den Berg et al., 2018; Lim et al., 2020; Morris et al., 2020). Generally, higher serum levels of TNF-α are linked to more severe CPLBP (Teodorczyk-Injeyan et al., 2019), radicular pain (Uceyler et al., 2007; Zu et al., 2016) and disability (Wang et al., 2016). Additionally, owing to its predictive capacity, urinary TNF-α may serve to discriminate between responders and non-responders in future clinical studies.

Biomarkers can also serve as indicators of recovery or predictors of treatment response (Khan et al., 2017; Davis et al., 2020). Our findings are compatible with urinary TNF-α being a potential biomarker to assess clinical recovery in this cohort of CPLBP patients. This holds particular relevance, as changes in both clinical outcomes may be considered clinically meaningful (Ostelo et al., 2008). Baseline values of urinary TNF-α explained 20.7% of the changes in pain intensity and 38.1% of the variance in disability scores following treatment. Likewise, the percent change in TNF-α predicted 65% and 33% of the changes in pain intensity and disability scores, respectively, suggesting its potential as a reliable, objective measure of treatment response. Similar data have not been reported thus far. However, in a cohort of elderly women with an acute episode of LBP, serum TNF-α levels decreased concurrently with reductions in LBP intensity over 12 months (Queiroz et al., 2017). Similarly, Klyne and colleagues observed that higher baseline TNF-α levels and depressive symptoms were associated with lower probability of recovery from acute LBP (Klyne et al., 2017, 2022; Klyne and Hodges, 2020). Thus, reduction in TNF-α levels may be indicative of recovery from episodes of LBP, which is consistent with our data. Alternatively, persistently elevated levels may be associated with a lack of recovery (Klyne et al., 2017, 2022) or with ongoing CPLBP symptoms with minor or major fluctuations, but without long pain-free periods. It could be argued that patients with persistent pain have higher levels of TNF-α consistent with no recovery, while patients with episodic CPLBP display the lowest levels, reflecting their capacity to recover from an episode.

It may be suggested that TNF-α could mediate neuroinflammatory changes associated with a subgroup of patients with a more severe CPLBP trajectory. Notably, TNF-α has been identified as an important cytokine for the development of changes in the central nervous system that lead to pain hypersensitivity and persistence (Andrade et al., 2011; Zhang et al., 2011; Cairns et al., 2015; Ji et al., 2018; Goncalves Dos Santos et al., 2019). Here, we hypothesized that TNF-α could serve as a biomarker for a subgroup of patients with CPLBP. Particularly, where neuroinflammation, and therefore, central sensitization exists. Previous attempts to classify patients with CPLBP according to pain mechanisms suggested three subgroups: nociceptive, neuropathic, and central sensitization pain (Smart et al., 2012; Nijs et al., 2015). However, there is no consensus on the clinical methods that can accurately discriminate between pain mechanisms. A recent systematic review highlights that urine metabolomics analysis is one of the most reliable measures identified to distinguish neuropathic pain mechanisms (Shraim et al., 2021), suggesting that urine could be a promising environment for pain biomarkers. Despite the limited range of available neuropathic pain biomarkers, serum levels of TNF-α have been demonstrated to be particularly effective in detecting neuropathic pain in patients with spinal cord injury (Xu et al., 2015). Given that the present study specifically excluded patients presenting evidence of neuropathic pain, it is plausible that elevated TNF-α may reflect processes related to central sensitization in individuals with both neuropathic and nociplastic pain (Carlton et al., 2009; Woolf, 2011; Nijs et al., 2021).

Biomarkers can also provide insights into the mechanisms of interventions (Davis et al., 2020). The results from the present study may contribute to our understanding of the potential mechanisms underpinning SMT for CPLBP. Higher baseline TNF-α was associated with better clinical recovery, suggesting that SMT may be more effective for a subgroup of patients with elevated TNF-α levels. This is congruent with literature suggesting that SMT may act by modulating mechanisms related to central sensitization (Gevers-Montoro et al., 2021). Nevertheless, no causal relationship can be inferred from the data and caution is advised when interpretating them.

### Limitations of the study

The discussed findings must be interpreted in light of a series of limitations, which include the lack of a control intervention group and the small sample size. As an observational study, changes cannot be attributed to the intervention or any other factors. Future experimental research with appropriate comparators may examine whether reductions in urinary TNF-α reflect a specific mechanism of SMT for CPLBP. A placebo-controlled design is also required to confirm previous findings suggesting that SMT dosage may influence plasma concentrations of inflammatory cytokines, including TNF-α (Licciardone et al., 2012; Duarte et al., 2022). Based on our data, an association between the total number of SM applied to the low back cannot be confirmed or excluded.

Additionally, this study’s categorization of CPLBP, acknowledged as a heterogeneous condition, inherently poses a risk of overgeneralization. The extent and predominance of nociplastic mechanisms likely differ among CPLBP patients, potentially affecting TNF-α expression and complicating the extrapolation of the study results. Furthermore, the limited sample size demands caution when interpreting the subgroup analyses. Prudence is warranted in light of recent evidence suggesting that patients’ recollection of their LBP pattern (episodic vs. fluctuating) using visual pain trajectories may not be as reliable as indicated by previous data (Nim et al., 2023). In future studies, longer follow-up periods may help determine whether changes in cytokines and clinical variables, and their association, persist over time. In addition, variables such as diet or exercise that were not accounted for, may have influenced systemic inflammation, and thus, TNF-α levels. Future research should take these and other potential confounders into consideration.

Urine samples were collected during different seasons for the CPLBP (January to April) and control (September to January) groups. Seasonal variations of serum TNF-α were reported in conditions with seasonal variability, though not for healthy individuals. The highest TNF-α values were observed during summer-fall, and the lowest from January to spring (Spath et al., 2017; Weckmann et al., 2021). This pattern is contrary to our findings, suggesting that seasonal variations may not have influenced the results. Despite the aforementioned limitations, a strength of this study lies in the advantages of urine sampling compared to the traditional serum sampling. It is plausible that using urine samples provides similar results with much greater accessibility, fewer logistic challenges and at a lower cost.

## Conclusion

This exploratory study presents evidence suggesting that urinary levels of TNF-α may serve as a potential biomarker for patients with CPLBP. Specifically, urinary TNF-α levels discriminated patients with CPLBP from pain-free controls in our sample. These results warrant further study to assess urinary TNF-α levels among patients with different pain trajectories. In addition, our findings indicated that baseline values and fluctuations in TNF-α could predict pain intensity and disability outcomes. Consequently, urinary TNF-α levels may potentially reflect the involvement of inflammatory mechanisms in CPLBP evolution, although this remains to be examined. Further research, preferably in the form of a randomized controlled trial, is needed to better ascertain the utility of this potential biomarker for CPLBP.

## Data availability statement

The raw data supporting the conclusions of this article will be made available upon reasonable request, without undue reservation.

## Ethics statement

The studies involving human participants were reviewed and approved by the Madrid College of Chiropractic Research subcommittee (San Lorenzo de El Escorial, Madrid, Spain) and the Fundación Jiménez Díaz Hospital Clinical Research Ethics Committee (Madrid, Spain). The patients/participants provided their written informed consent to participate in this study. Written informed consent was obtained from the individual(s) for the publication of any potentially identifiable images or data included in this article.

## Author contributions

CG-M: conceptualization, methodology, investigation, formal analysis, and writing—original draft preparation. MP-T and AM: investigation, formal analysis, and manuscript revision. FC-B: investigation, resources, and manuscript revision. MP: supervision, funding acquisition, and writing–review and editing. AO-D: conceptualization, methodology, funding acquisition, supervision, writing–review and editing, and approval of final version. All authors contributed to the article and approved the submitted version.
