# Supplementary material for: Urinary TNF-α as a potential biomarker for chronic primary low back pain
Source: Front Integr Neurosci. 2023 Jun 28;17:1207666. doi: 10.3389/fnint.2023.1207666 (PMC10336221; doi:10.3389/fnint.2023.1207666)
Supplement: Supplementary file 1 [file Data_Sheet_1.docx]

**Supplemental Figure S1.** Heatmap of Spearman rank correlations between follow-up and percent changes in TNF-α and the number of manipulations targeting different segments. Values in the boxes represent Spearman coefficient ρ.


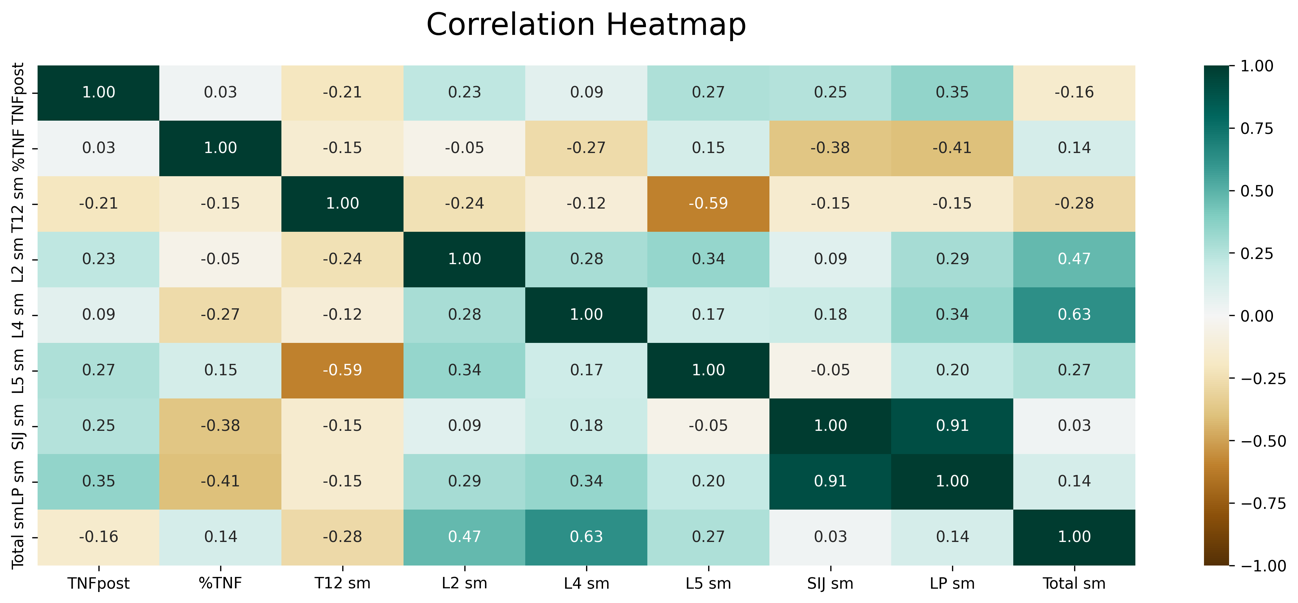


*‘TNFpost’: Follow-up levels of TNF-α; ‘%TNF’: Percent changes in TNF-α; ‘T12 sm’: number of spinal manipulations targeting T12; ‘L2 sm’: number of spinal manipulations targeting L2; ‘L4 sm’: number of spinal manipulations targeting L4; ‘L5 sm’: number of spinal manipulations targeting L5; ‘SIJ sm’: number of spinal manipulations targeting the sacroiliac joints; ‘LP sm’: number of spinal manipulations targeting the lumbopelvic spine; ‘Total sm’: total number of spinal manipulations applied.*
